# Protective Effect of Deer Heart Peptide on Cardiac Injury in Mice

**DOI:** 10.1155/2024/6661371

**Published:** 2024-05-14

**Authors:** Qun Zhang, Hongjin Li, Dongshu Jia, Wei Chen, Xinao Jia, Qi Lin, Jiyi Zhang, Yujiao Tang

**Affiliations:** ^1^School of Life Sciences, Changchun Sci-Tech University, Changchun 130600, Jilin, China; ^2^Department of Food Science and Nutrition, College of Health Science, Dong-A University, Busan 49315, Republic of Korea; ^3^Jilin Sino-ROK Institute of Animal Science, Changchun 130600, Jilin, China; ^4^School of Vocational and Technical Education, Changchun Sci-Tech University, Changchun 130600, Jilin, China; ^5^College of Food Science and Engineering, Jilin Agricultural University, Changchun 130118, Jilin, China

## Abstract

Peptides are widely used as natural bio-small molecules because of their various pharmacological activities such as enhancing immunity, promoting wound healing, and improving inflammation. Alcoholic heart injury has become one of the major health problems worldwide, and alcohol consumption is now the main cause of alcoholic cardiomyopathy. In this study, deer heart peptides were extracted from deer hearts by enzymatic digestion and the antioxidant activity of deer heart peptides extracted at different times was evaluated by three in vitro antioxidant methods, and the active peptide with the best enzymatic effect has been selected for in vivo animal experiments. The anti-inflammatory and antioxidant properties of deer heart enzymatic extracts were evaluated in in vivo experiments in mice. In this study, mice were orally gavaged with white wine (12 mL/kg body weight) to induce a mouse model of cardiac injury, while mice were orally administered a single dose of 100 mg/kg/bw and 200 mg/kg/bw of deer heart enzyme digest and were examined for body weight, dietary intake, water intake, and coat gloss, as well as for general behaviors, adverse effects, and mortality. Histology, serum, anti-inflammatory factors, and oxidative stress parameters were subsequently assessed. In all modeled mice, no four-way or any significant behavioral changes were observed in all groups, but in the modeled group, mice showed weight loss, decreased diet and water intake, and decreased cardiac index. For in vivo tests, the extract inhibited the anti-inflammatory activity with a significant decrease in inflammatory factors of TNF-*α*, IL-6, and IL-1*β* in cardiac tissues, a significant increase in serum levels of both CAT and SOD, an increase in MDA content, and a remarkable increase in the level of the marker CK in the cardiac myocardial enzyme profile. Significant improvement in myocardial disorders by deer heart peptide could be observed from heart tissue sections. The present study emphasizes the anti-inflammatory and antioxidant activity of deer heart peptide, an enzymatic digest of deer heart, which provides empirical as well as supportive role for the anti-inflammatory properties of traditional medicine.

## 1. Introduction

*Cervus nippon* is a valuable medicinal and animal-based food in China, and its body is full of treasures and extremely rich in bio actives [1]. China is the first country in the world to breed and produce *C. nippon*, and with the development and progress of the times, many countries in the world, such as Japan, Korea, and the United Kingdom, have also started to breed *C. nippon* on a large scale and develop and utilize its related functional products [2, 3]. Products recovered from *C. nippon* are well known for its health promising properties, thus identified as valuable medicinal herbs at home and abroad. Its deer heart, deer blood, and antler blood are the hot spots for the development of plum deer resources in China in recent years [4]. Deer hearts mostly refers to the heart of plum deer or horse deer [5], which is rich in a variety of amino acids, vitamins, peptide proteins, and other substances and is a good medicine to enhance the metabolic function of human body and improve cardiovascular diseases and nervous system functions [6]. As far as our traditional Chinese medicine theory is concerned, the tissues and organs of animals have protective, preventive, and therapeutic effects on the corresponding tissues and organs of the human body [7]. In recent years, it has been shown that small molecule active peptides from deer heart can regulate cardiac dysfunction, promote blood circulation, and replenish myocardial prime mover, thus achieving a protective effect on the heart [8].

Peptides are protein fragments formed by peptide bonds connecting two or more amino acid molecules and are biologically active substances with multiple cellular functions. Peptides are classified into dipeptides, tripeptides, and polypeptides according to amino acid residues, and they are a class of protein precursors or degradable products between proteins and amino acids [9, 10]. In recent years, peptides have become popular among domestic and foreign researchers owing to their small molecular weight, easy absorption by the body, high activity, and low side effects [11]. According to the available studies, peptides have various functional characteristics such as immunomodulation, antioxidant, hypotensive, antibacterial, and antiviral [12–15].

Dong [16] et al. have pointed out that the chemical composition of plum deer heart is complex and has revealed that it contains a variety of physiologically active substances, and its products can significantly increase coronary blood flow in rats under specific experimental conditions. It showed that the heart of the plum deer has a significant protective effect on the cardiovascular system. Zhao [17] et al. showed by UPLC analysis that the enzymatic digest of deer heart contained 17 amino acids, the content of which was higher than that of deer heart extract without enzymatic treatment, and the nutritional value was higher. Chen [18] et al. demonstrated that deer heart small molecule active peptides significantly reduced the release of creatine kinase (CK), aspartate transaminase(AST), and LDH-L after H/R injury; decreased the content of lipid peroxide metabolite malondialdehyde (MDA); increased the SOD activity of cardiomyocytes; inhibited the expression of inflammatory factors tumor necrosis factor-*α* (TNF-*α*), interleukin-6 (IL-6), and interleukin-1*β* (IL-1*β*) in cardiomyocyte cultures; and had a protective effect on cultured cardiomyocytes in vitro.

The proper functioning of the heart depends on a timely and adequate supply of blood from the coronary arteries. When the blood supply to the heart is inadequate, cardiac ischemia or myocardial hypoxia can occur. Myocardial ischemia or hypoxia can lead to cellular metabolic disorders, cellular damage, and thus heart dysfunction, which can lead to many heart diseases [19]. Cardiovascular disease is the main disease that endangers human life, and its incidence is increasing year by year [20]. Currently, alcohol consumption is the main cause of dilated cardiomyopathy in China, and dilated cardiomyopathy caused by alcohol consumption, also known as alcoholic cardiomyopathy, is also common in clinical practice. The heart is damaged, causing ischemia and hypoxia in the myocardial cells of the blood supply area, resulting in myocardial cell necrosis. Cardiomyocytes are the basic units that make up the heart and are contractile and diastolic. Alcohol consumption leads to fibrosis of cardiomyocytes and interstitial myocardium, making the myocardium less systolic and diastolic. Studies have shown that excessive alcohol consumption can cause heart failure and irreversible damage to the heart muscle. Once alcoholic cardiomyopathy occurs, besides causing severe heart failure, it can cause serious arrhythmias, which pose a serious threat to human health and have become urgent concerns [21].

At present, most of the research reports on peptide extraction from deer products focus on antler, deer blood, and deer whip. In spite of this, few studies have been published on the extraction of peptides from deer hearts. Through the search of corresponding literature, it was found that there are few systematic data reported about the antioxidant, anti-inflammatory, and preventive treatment of deer heart in heart diseases. In order to investigate the effects of deer heart peptide on populations with potential risk factors for diseases such as heart injury and potential therapeutic targets, we established a mouse model of alcoholic heart injury by using a high concentration of edible alcohol and then administered different concentrations of deer heart peptide to the mice by transoral gavage for subsequent testing. In this study, we investigated the effects of deer heart peptide on alcohol-induced cardiac injury in mice and explored the potential mechanisms and relationships between the antioxidant activity, anti-inflammatory properties, and cardiac injury of deer heart peptide *in vivo* and *in vivo*. Therefore, the study of using deer heart peptide on mouse heart injury model not only provides theoretical basis for the development and utilization of bioactive peptides but also lays the theoretical foundation for the development of heart nutritional supplements and treatment of cardiovascular diseases using deer heart peptide as raw material.

## 2. Methodology

### 2.1. Drugs and Chemicals

The deer heart was originally purchased from Jilin Dong'ao Deer Technology Development Co. Ltd. and extracted and purified to deer heart peptide by Jilin China-Korea Institute of Animal Science. Chemicals used in this study were purchased from local commercial stores from the corresponding suppliers.

### 2.2. Sample Preparation

We precisely weighed 1g of deer heart tissue and pepsin was added at 1% with a solid-liquid ratio = 1 : 100 and enzymatically digested for 3 h, 5 h, 7 h, and 9 h at 37°C. Subsequently, the samples were centrifuged and filtered, evaporated, and concentrated; after freezing, the product is lyophilized. Through the results of in vitro antioxidant, the 7 h digested deer heart peptide was finally selected for the subsequent in vivo experiments in mice, taking into account the economic factors and the smooth running of the experiments.

### 2.3. Activation of Antioxidants

#### 2.3.1. Scavenging of Radicals by DPPH

A slightly modified version of the Blois method was used to measure each deer heart peptide's DPPH scavenging activity [22]. The DPPH solution (1.5 × 10^−4^ M, 100 *μ*L) was mixed with and without each extract (100 *μ*L) and incubated at room temperature for 30 minutes. After 30 minutes of standing, with the aid of an enzyme marker, the absorbance was measured at 540 nm. Using the following equation, clearance activity was calculated as a percentage.

(1) $$\begin{array}{c} \text{Inhibition}\left(\%\right)=\frac{\left(A_{\text{control}}-A_{\text{sample}}\right)}{A_{\text{control}}}. \end{array}$$

An absorbance measurement of the reaction mixture without deer heart peptide is used as *A*_control_ and deer heart peptide absorbance *A*_sample_ is determined by the reaction mixture containing the sample.

### 2.4. Hydrogen Peroxide Radical Scavenging Activity

As determined by Muller's method, hydrogen peroxide scavenging activity was calculated [23]. In 96-microtiter plates, 100 *μ*L of 0.1 M phosphate buffer (pH 5.0) was added to each extract. After 5 minutes of incubation at 37°C, 20 *μ*L of hydrogen peroxide was added to the mixture. A mixture of 30 *μ*L of ABTS containing 1.25 mM and 30 *μ*L of peroxidase containing 1 unit/mL is then added to the mixture and incubated for 10 minutes at 37°C. In order to calculate the percentage of scavenging activity, a 405 nm enzyme marker was used to measure absorbance and a scavenging activity percentage was determined by using (1).

### 2.5. ABTS Free Radical Scavenging Activity

ABTS scavenging activity of deer heart peptides enzymatically digested at different times was assessed according to the method of Chung et al. [24]. A solution of ABTS-+ and a solution of potassium persulfate were used as stock solutions. Mixing equal amounts of the 2 stock solutions and allowing them to react for 12 hours prepared the working solutions. The working solution was diluted with fresh ABTS-+ solution and mixed with or without extracts. Two-hour incubation was followed by the measurement of each solution's absorbance at 735 nm. As a result of (1), a scavenging activity percentage was calculated.

### 2.6. Animals and Diets

Eight-week-old male C57BL/6 mice weighing 23 ± 03 g were purchased from an approved laboratory animal supplier. In a 12-hour light/12-hour dark cycle, the animals were housed with thermoregulation (22 ± 1°C) and humidity (40 ± 10%). Mice are acclimated to the laboratory environment for one week prior to the test. The animals are fed a standard rodent diet and received free access to purified water (reverse osmosis autoclaved water).

### 2.7. Alcoholic Cardiac Injury Induction

Treating mice with alcohol resulted in alcohol-induced heart disease with 60° white wine for 1 week (12 mL/kg body weight). During the establishment of the alcoholic heart injury model, there was a 90% success rate and no animals died during the experiment, which can be used for further studies.

## 3. Experimental Design

**Figure 1:**
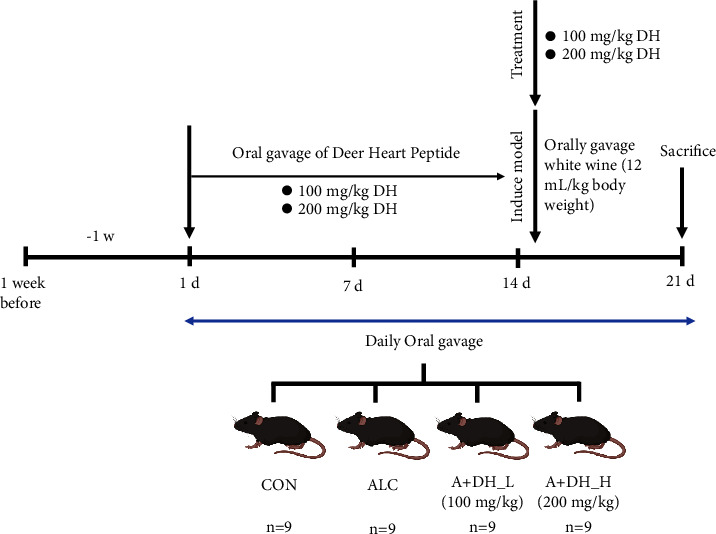
Adaptive feeding for one week prior to oral administration. Subsequently, they were intervened with daily oral administration of deer heart peptide (DH: 100 and 200 mg/mL) for 21 days. White wine (ALC: 12 mL/kg) was administered orally daily for 6 days starting from day 15 to induce alcoholic heart injury.

Animals were randomly divided into four groups of nine mice each. Following is a list of the groups: Group I: control group—normal diet with water; Group II: alcoholic heart injury model group—received white wine by oral gavage; Group III: model mice received 100 mg/kg of deer heart peptide; Group IV: model mice received 200 mg/kg of deer heart peptide for 21 days. The deer heart peptide was dissolved in distilled water. The mice in the model group and the administered group were given an equal amount of distilled water along with transoral gavage in the control group (Figure 1). Subsequently, they were intervened with daily oral administration of deer heart peptide (DH; 100 and 200 mg/mL) for 21 days. White wine (ALC; 12 mL/kg) was administered orally daily for 6 days starting from day 15 to induce alcoholic heart injury.

All samples were dissolved in deionized water and administered orally once a day for 21 days. Mice were fasted and executed after 21 days.

Blood was collected for the next step of analysis. Mice were weighed, and mouse hearts were collected, frozen in liquid nitrogen, and stored at −80°C until further study, and heart tissues were also fixed in 10% formalin for the next histological analysis.

Ethical approval for this study was obtained from Changchun Sci-Tech University (CKARI202302).

### 3.1. Weight, Food Intake, Water Intake, and Cardiac Weight Index Are Assessed Each Week

Food intake, water intake, and body weight of the animals were recorded every other day until the end of the experimental period. The absolute weight of the heart was recorded on the day of dissection, and its relative weight was calculated using the formula below [25].

(2) $$\begin{array}{c} \text{Heart mass index }\left(\text{HWI}\right)=\text{mouse wet heart weight }\left(\text{mg}\right)/\text{mouse body weight }\left(g\right). \end{array}$$

### 3.2. The Detection of Cardiomyocyte Injury Markers and Markers of Oxidative Stress

Blood samples were obtained by cardiac puncture and serum was separated by centrifugation (3000 rpm, 20 min) and stored at −80°C until assay. Creatine kinase isoenzyme (CK) concentrations were assessed as indicators of cardiomyocyte injury. Superoxide dismutase (SOD) activity, catalase (CAT), and malondialdehyde (MDA) levels were used as indicators of oxidative stress. CK levels in serum cardiac tissues and markers of oxidative stress in cardiac tissues were measured by commercially prepared kits (Nanjing Jincheng Institute of Biological Engineering, Nanjing, China).

### 3.3. Histological Analysis

An aqueous solution was applied to the heart tissue to fix it in a phosphate buffer solution that contained 10% formalin. The dehydrated tissue was dipped in wax, embedded and cut into 5 *μ*m thick, thin pieces, and stained with hematoxylin and eosin (H&E). The heart samples were observed and photographed (×100 original magnification) under a microscope.

### 3.4. Evaluation of the Transcript-Level Expression of Target Genes Using Quantitative Real-Time PCR (qPCR)

Total RNA was isolated from heart tissues of mice belonging to each treatment using TRIzol according to the manufacturer's protocol. cDNA was obtained using Superscript II switch transcriptase (Invitrogen). cDNA was analyzed by RT-qPCR on Warm Cycler Shakers TP850 (Takarabio Inc., Shigatse, Japan) according to the manufacturer's protocol. Briefly, 2 *μ*L of cDNA (100 ng), 1 *μ*L of sense and antisense primers (0.4 *µ*M), 12.5 *μ*L of SYBR Premix Ex Taq (Takarabio Inc.), and 9.5 *μ*L of dH_2_O were mixed to obtain 25 *μ*L of solution. PCR primers used for gene expression analysis are listed in Table 1. The amplification conditions were as follows: 95°C for 10 s, 95°C for 5 s, 60°C for 30 s, 95°C for 15 s, 60°C for 30 s, 95°C for 15 s, and 40 cycles of C. Primers for qPCR were synthesized by Enotech Co. (Daejeon, Korea) (Table 1). The target gene expression was normalized to that of glyceraldehyde 3-phosphate dehydrogenase using GAPDH as an internal reference gene, and the relative mRNA expression of the target gene was determined using the 2^−ΔΔCt^ method.

### 3.5. Statistical Analysis

One-way ANOVA was used to determine differences between groups, and data were expressed as mean and standard error. A post hoc test for Turkey was performed using GraphPad Prism software (v.8.0; GraphPad Software, La Jolla, CA, USA); *P* < 0.05 was considered significant.

## 4. Results

### 4.1. Antioxidant Activity

The antioxidant activity of deer heart peptides may not be attributed to a single mechanism. Therefore, in this study, three methods were chosen to assess different aspects of the antioxidant activity of deer heart peptides.

**Figure 2:**
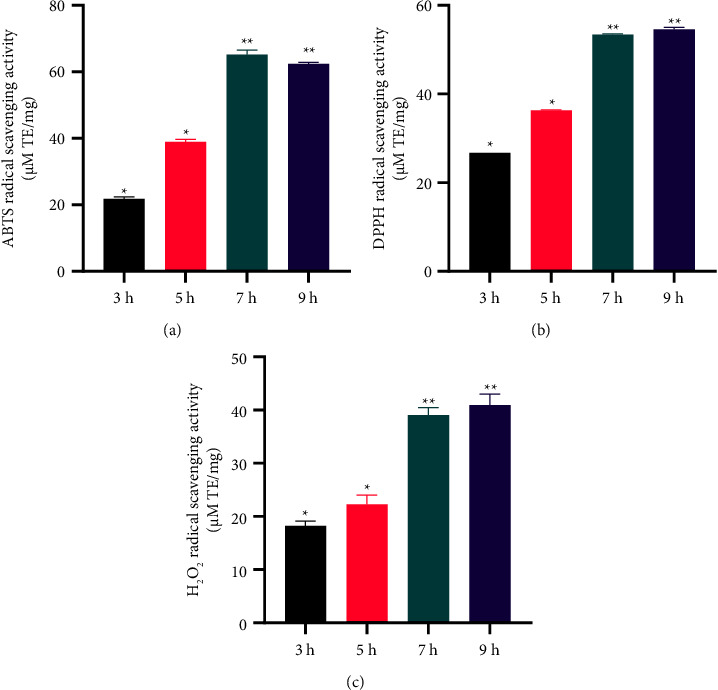
A comparison of the antioxidant activity of deer heart peptides. (a) ABTS radical scavenging activity of deer heart peptides enzymatically digested at different times. (b) DPPH radical scavenging activity of deer heart peptides enzymatically digested at different times. (c) H_2_O_2_ radical scavenging activity of deer heart peptides enzymatically digested at different times. Data represent mean ± standard error of the mean. ^*∗∗*^*p* < 0.05; ^*∗*^*p* < 0.01 according to Dunnett's multiple range test.

*In Vitro Antioxidant Assay*. The antioxidant activity of the enzymatic extracts of deer heart was assessed by ABTS, DPPH, and H_2_O_2_ assay and is shown in Figure 2. Overall, the antioxidant activity of the 9 h enzymatic digestate of deer heart peptide was markedly better than that of deer heart peptide of 3 h, 5 h, and 7 h enzymatic digestate and appeared to be dose dependent. From the ABTS results, the antioxidant activity of 7 h deer heart enzyme digestate (65.1505 *μ*M TE/mg, Figure 2(a)) was higher than that of 9 h deer heart peptide (62.336 *μ*M TE/mg, Figure 2(a)), but there was no significant difference between them (*P* < 0.01). The antioxidant activity measured with DPPH (54.5875 *μ*M TE/mg, Figure 2(b)) and H_2_O_2_ (40.929 *μ*M TE/mg, those of 7 h deer heart peptides, with no significant differences (*P* < 0.01), Figure 2(c)). Therefore, it was decided to use the 7 h deer cardiac peptide enzymatic digestate for the subsequent experimental study based on a combination of factors such as economy and smoothness of the experimental conduct.

### 4.2. Effects of Deer Heart Peptide on Body Weight, Food Intake, Water Intake, and Heart Weight of Mice with Liquor-Induced Alcoholic Heart Injury

**Figure 3:**
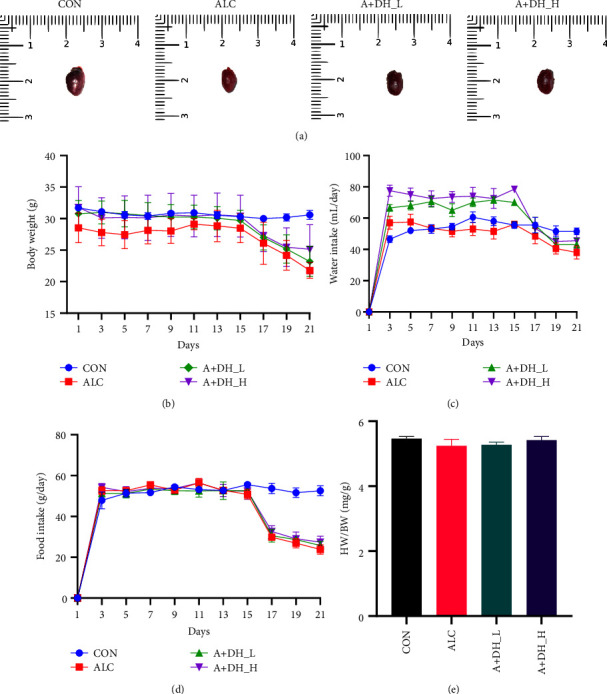
Effects of deer heart peptide on body weight, food intake, water intake, and heart weight. (a) Heart morphology in mice with alcoholic heart injury. (b) Food intake. (c) Water intake. (d) Heart weight/body weight (HW/BW) of mice with alcoholic heart injury. (e) Cardiac index in mice. CON: normal control mice; ALC: alcoholic heart injury; A + DH_L: mice with alcoholic heart injury treated with 100 mg/kg/bw of deer heart peptide; A + DH_H: mice with alcoholic heart injury treated with 200 mg/kg/bw of deer heart peptide.

Figure 3(a) illustrates the morphological map of the heart of each group of mice.  Figure 3(b) depicts the weight changes of mice in the control and experimental groups every two days. During the first 15 days of oral administration of deer heart peptide, the body weight of mice in both the normal diet group and the deer heart peptide-fed group stabilized. However, after the 15th day, the white wine modeling phase was performed, and the mice fed orally with white wine lost the most significant weight compared to the mice fed with normal diet. In contrast, after deer heart peptide administration (100 and 200 mg/mL body weight), body weight decreased but the decrease was alleviated compared to the model group, and the decrease was slower in the deer heart peptide at high dosage (Figure 3(b)). Figures 3(c) and 3(d) depict the changes of diet and water consumption of mice during the experimental period, and the trends of both were largely the same, with a significant decrease in diet and water consumption after oral gavage of white wine. The heart weight/body weight of the experimental animals did not show any significant changes compared with the control animals (Figure 3(e)), but the heart weight index of the mice in the deer heart peptide tended to approach that of the control group.

### 4.3. Effect of Deer Heart Peptide on the Pathological Morphology of Alcoholic Heart Injury

**Figure 4:**
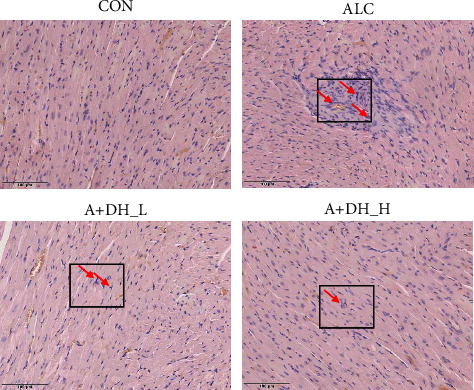
Effect of deer heart peptide on the pathological pattern of alcoholic heart injury. Tissue sections were photographed under a microscope (original magnification ×100). CON, control group; ALC, alcoholic heart injury model group; A + DH_L, supplemented with low-dose deer heart peptide 100 mg/kg body weight/day; A + DH_H, supplemented with high-dose deer heart peptide 200 mg/kg body weight/day.

A representative H&E-stained tissue section of the heart is shown in Figure 4. In the H&E staining, the nuclei are blue and the collagen fibers appear red. In the CON group, myocardial fibers was arranged in neat rows showing myocardial cells without atrophy or hypertrophy, indicating normal myocardial structure, while in the ALC group, disorganized myocardial fibers with severe damage and loss of normal ordered structure could be clearly seen. The number of disordered myocardial fibers was reduced or even disappeared in the A + DH_L and A + DH_H groups compared with the ALC group. Meanwhile, it is more visualized from the figure that the cardiomyocytes in the ALC group had obvious swelling and gradual disappearance of transverse lines. However, after deer heart peptide prophylaxis, the cardiomyocytes gradually decreased in size and appeared cross-striations, which was most obvious in the high-dose group. The results indicate that deer heart peptide can reduce the degree of cellular disorder in heart tissue and has a protective effect on the heart.

### 4.4. Effects of Deer Heart Peptide on the Oxidative Status of the Heart and Markers of Cardiomyocyte Damage in Mice with Alcohol-Induced Alcoholic Heart Injury

**Figure 5:**
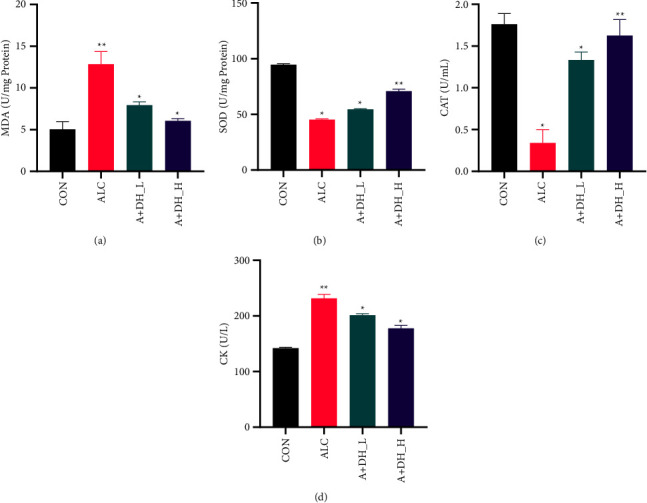
Deer heart peptide administration affects SOD activity, MDA content, CAT activity, and CK levels. (a) Malondialdehyde (MDA) levels in myocardial tissues of control group, ALC group, and deer heart peptide-treated mice with liquor-induced heart injury. (b) Superoxide dismutase (SOD) levels in myocardial tissues of control, ALC, and deer heart peptide-treated White wine-induced mice. (c) Catalase (CAT) levels in myocardial tissues of control, ALC, and deer heart peptide-treated White wine-induced mice. (d) Creatine kinase (CK) levels in serum of control, ALC, and deer heart peptide-treated liquor-induced mice. Data represent mean ± standard error of the mean. ^*∗∗*^*p* < 0.05; ^*∗*^*p* < 0.01 according to Dunnett's multiple range test. CON: control group; ALC: ALC-treated group; A + DH_L; 100 mg/mL deer heart peptide; A + DH_H; 200 mg/mL deer heart peptide.

The present study revealed changes in the oxidative stress response as well as cardiac serum markers in mice with alcoholic heart injury. The ALC group exhibited a significant increase in MDA (*P* < 0.01) (Figure 5(a)) levels and a significant decrease in SOD activity (*P* < 0.01) (Figure 5(b)), compared to the control group. In contrast, mice with alcoholic heart injury treated with 100 mg/mL and 200 mg/mL of deer heart peptide showed significant improvement in reduced MDA levels as well as increased SOD antioxidant enzyme activity. It can be seen from the CAT activity (Figure 5(c)) that the deer heart peptide supplement can improve the antioxidant activity in the body, and the value of the high-dose group is close to that of the CON group. And compared with the model group, the deer heart peptide supplement group had a significant increase (*P* < 0.05). Serum levels of CK were significantly (*P* < 0.05) increased in alcoholic heart-injured mice compared to that in control group (Figure 5(d)). Treatment of mice with 100 mg/kg (*P* < 0.05) or 200 mg/kg (*P* < 0.05) of deer heart peptide resulted in a significant decrease in CK levels compared to mice with alcoholic heart injury, with a significant difference.

### 4.5. Inflammatory Effects of Deer Heart Peptide on Alcohol-Induced Heart Damage

**Figure 6:**
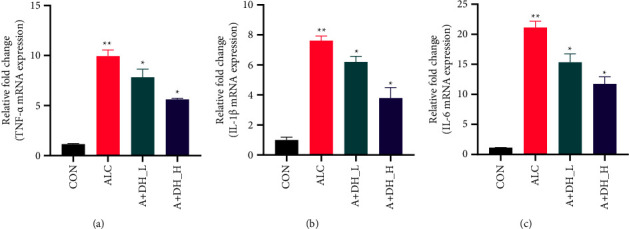
Effects of deer heart peptide administration on TNF-*α*, IL-1*β*, and IL-6 inflammatory cytokines. Quantitative real-time qPCR analysis of mRNA levels in mouse heart tissue. The graphs show the mRNA expression of (a) TNF-*α*, (b) IL-1*β*, and (c) IL-6. Data represent mean ± standard error of the mean. ^*∗∗*^*P* < 0.05; ^*∗*^*P* < 0.01 according to Dunnett's multiple range test.

The abnormal proinflammatory response leads to alcohol-induced cardiac injury. Therefore, to more clearly characterize the alcohol-induced cardiac-associated inflammatory response, the mRNA expression of the proinflammatory cell markers tumor necrosis factor a (TNF-*α*), interleukin 1*β* (IL-1*β*), and Interleukin 6 (IL-6) was significantly upregulated (relative to the CON group) in mice with alcoholic heart injury (*P* < 0.05). And as clearly seen in the figure (Figures 6(a)–6(c)), deer heart peptide pretreatment effectively reduced the three indices (*P* < 0.01), indicating that deer heart peptide effectively reduced cardiac inflammation in alcoholic heart-injured mice. All these data suggest that deer heart peptide prevents alcohol-induced cardiac injury by inhibiting the overproduction of proinflammatory cytokines.

## 5. Discussion

With the growing concern of consumers towards the adverse effects associated with synthetic compounds, researchers focused on the exploration of natural remedies for many diseases. *Cervus nippon* has played a central role in traditional Chinese medicine owing to the promising bioactive compounds present. Therefore, in the current study, we examined the protective effect of deer heart peptide recovered with enzymatic digestion on alcoholic heart-injured mice. Our findings revealed the promising effect of deer heart peptide on alleviating the clinical features of alcoholic heart-injured mice [26–28]. In the present study, we created an alcoholic heart-injured mice model through the administration of white wine and increased levels of serum SOD, MDA, and CK confirmed that alcoholic heart-injured mice model was successfully created. In addition, clinical characteristics such as weekly body weight, food intake, heart weight, and heart weight/body weight demonstrated that the creation of a mouse model of alcoholic cardiac injury was successful. Similar results were reported with the previous study where reduced heart weight and serum cardiac markers were the prominent features of heart-injured mice [29].

Cardiac injury is a common clinical cardiovascular disease, and its incidence has been increasing year by year in recent years. Therefore, it is urgent to solve this problem. Xu [30] et al. used velvet polypeptide as a pharmacodynamic substance to observe its protective effect on mouse heart injury and found that velvet has various functions such as antioxidation and promoting protein and nucleic acid synthesis in vivo. Cardiomyocyte-specific transcription factors (Nkx2.5, GATA4, ATF-2, and MEF-2C) have a significant inhibitory effect on cardiac injury. Therefore, it can be speculated that deer heart peptide has good antioxidant activity, can activate various proteins that regulate the heart muscle, and has a protective effect on the heart. Modern pharmacological studies have shown that the protein content in velvet antler is as high as 55.26%. Zhao et al. [31] confirmed that velvet antler protein can significantly reduce the damage changes of the electrocardiogram, reduce the area of myocardial ischemia, and reduce myocardial fibrosis. In summary, we can guess that the protein in deer heart peptide can reduce heart injury in mice. In addition, Chen et al. [32] further confirmed the protective effect of pilose antler polypeptide on rat heart injury and observed its effect on SOD activity in ischemic myocardial tissue. Therefore, we can speculate that peptides have good antioxidant activity, which can reduce the generation of free radicals in the body and increase SOD activity.

Deer heart peptide is highly antioxidant, which means that it activates the body's antioxidant capacity, increases SOD activity, and reduces MDA production in the body. The degree of cardiomyocyte damage was measured by CK and H&E staining, which is a marker enzyme for the degree of cardiac damage, while H&E staining allows visualization of the degree of myocardial disorder in mice to count the size of the cells. Apparently, deer heart peptide can successfully reduce the level of CK in vivo and can reduce cardiac fiber disorders and inhibit cardiomyocyte swelling in mice, which confirms that it has a wide therapeutic potential and has a series of prospects for development in the near future. However, this study was conducted to induce acute heart injury in mice by white wine, so it was more impaired to the body function and affected the organ index of mice. Therefore, it is necessary to pay attention to the loss of body function in mice in future studies, and it is needed to maintain the body function as much as possible while conducting effective studies. Therefore, further studies are needed in order to explore the possible mechanism of action of deer heart peptide and make it a useful clinical treatment.

The ancient literary works provide evidence of the favorable impact of deer heart on cardiac well-being, thereby highlighting its potential therapeutic significance. In contrast, there exist a plethora of scholarly investigations into the diverse medicinal properties of deer antler velvet, leaving deer heart as a relatively neglected subject of scientific inquiry [33, 34]. Consequently, this valuable organ, considered a byproduct of the deer industry, frequently falls victim to wastage. Compared to deer antler, deer heart exhibits a more modest price point in the market. Compared to antler, deer heart exhibits a smaller price in the market. Deer heart has great potential as a functional food in the health care industry, and in future studies, small molecule active peptides from deer heart can be used to explore related heart diseases such as myocarditis, myocardial infarction, coronary artery disease, and angina pectoris to investigate the efficacy of deer heart peptide and the related mechanism and to further confirm the ameliorative effect of deer heart peptide on heart injury. However, its intrinsic value as a functional food surpasses mere monetary considerations. Rich in bioactive compounds and potentially possessing unique physiological effects, deer heart holds promise as a natural source of health-promoting substances [18]. The integration of deer heart into the realm of functional foods can not only diversify the agricultural landscape but also enhance the economic prospects of farmers. By recognizing and utilizing the untapped potential of deer hearts, rural communities engaged in deer farming can expand their sources of income. In turn, it offers a compelling incentive for the growth and development of the deer industry, bolstering employment opportunities and local economies. Moreover, the sustainable utilization of deer heart aligns with the principles of resource conservation, promoting ecological balance within the agricultural sector.

In conclusion, the underappreciated deer heart, in light of its documented health benefits and comparative affordability, possesses immense potential as a value-added agricultural product. By elevating its status through scientific research, appropriate marketing channels, and consumer education, deer heart can emerge as a sought-after ingredient in the realm of functional foods, providing a tangible pathway for farmers to enhance their income and invigorate the burgeoning deer industry.

## Figures and Tables

**Table 1:** Primers used for qPCR.

Gene name		Sequence
TNF-*α*	Forward	5′-AAG CCT GTA GCC CAC GTC GT-′3
	Reverse	5′-GGC ACC ACT AGT TGG TTG TC-′3

IL-1*β*	Forward	5′-AAC CAA GCA ACG AVA AAA TA-′3
	Reverse	5′-AGG TGC TGA TGT ACC AGT TG-′3

IL-6	Forward	5′-CCG GAG AGG AGA CTT CAC AG-′3
	Reverse	5′-GGA AAT TGG GGT AGG AAG GA-′3

## Data Availability

The original contributions presented in the study are included in the article. Further inquiries can be directed to the corresponding author.

## Abbreviations

*C. nippon*: *Cervus nippon*
DH: Deer heart peptide
ALC: Alcoholic heart injury
A + DH_L: Mice with alcoholic heart injury treated with 100 mg/kg/bw of deer heart peptide
A + DH_H: Mice with alcoholic heart injury treated with 200 mg/kg/bw of deer heart peptide.
